# Surviving septic patients endotyped with a functional assay demonstrate active immune responses

**DOI:** 10.3389/fimmu.2024.1418613

**Published:** 2024-10-14

**Authors:** Adam D. Price, Ellen R. Becker, Evan L. Barrios, Monty B. Mazer, Patrick W. McGonagill, Christian B. Bergmann, Michael D. Goodman, Robert W. Gould, Mahil Rao, Valerie E. Polcz, Tamara A. Kucaba, Andrew H. Walton, Sydney Miles, Julie Xu, Muxuan Liang, Tyler J. Loftus, Philip A. Efron, Kenneth E. Remy, Scott C. Brakenridge, Vladimir P. Badovinac, Thomas S. Griffith, Lyle L. Moldawer, Richard S. Hotchkiss, Charles C. Caldwell

**Affiliations:** ^1^ Department of Surgery, University of Cincinnati College of Medicine, Cincinnati, OH, United States; ^2^ Sepsis and Critical Illness Research Center, Department of Surgery, University of Florida College of Medicine, Gainesville, FL, United States; ^3^ Department of Pediatrics, Case Western Reserve University School of Medicine, Cleveland, OH, United States; ^4^ Department of Surgery, University of Iowa Carver College of Medicine, Iowa City, IA, United States; ^5^ Department of Anesthesiology, University of Minnesota Medical School, Minneapolis, MN, United States; ^6^ Department of Pediatrics, University of Iowa Carver College of Medicine, Iowa City, IA, United States; ^7^ Department of Urology, University of Minnesota Medical School, Minneapolis, MN, United States; ^8^ Department of Anesthesiology, Washington University School of Medicine, St. Louis, MO, United States; ^9^ Department of Biostatistics, University of Florida College of Medicine, Gainesville, FL, United States; ^10^ Department of Surgery, Harborview Medical Center, University of Washington School of Medicine, Seattle, WA, United States; ^11^ Interdisciplinary Program in Immunology, University of Iowa Carver College of Medicine, Iowa City, IA, United States; ^12^ Department of Pathology, University of Iowa Carver College of Medicine, Iowa City, IA, United States; ^13^ Center for Immunology, University of Minnesota Medical School, Minneapolis, MN, United States; ^14^ Minneapolis VA Healthcare System, Minneapolis, MN, United States

**Keywords:** critical illness, late mortality, procalcitonin, IL-6, prediction modeling

## Abstract

**Introduction:**

Sepsis is a complex clinical syndrome characterized by a heterogenous host immune response. Historically, static protein and transcriptomic metrics have been employed to describe the underlying biology. Here, we tested the hypothesis that *ex vivo* functional TNF expression as well as an immunologic endotype based on both IFNγ and TNF expression could be used to model clinical outcomes in sepsis patients.

**Methods:**

This prospective, observational study of patient samples collected from the SPIES consortium included patients at five health systems enrolled over 17 months, with 46 healthy control patients, 68 ICU patients without sepsis, and 107 ICU patients with sepsis. Whole blood was collected on day 1, 4, and 7 of ICU admission. Outcomes included in-hospital and 180-day mortality and non-favorable discharge disposition defined by skilled nursing facility, long-term acute care facility, or hospice. Whole blood ELISpot assays were conducted to quantify TNF expression [stimulated by lipopolysaccharide (LPS)] and IFNγ expression (stimulated by anti-CD3/CD28 mAb), which were then used for assignment to one of four subgroups including an ‘immunocompetent’, ‘immunosuppressed endotype’, and two ‘mixed’ endotypes.

**Results:**

Whole blood TNF spot-forming units were significantly increased in septic and CINS patients on days 4 and 7 compared to healthy subjects. In contrast, TNF expression per cell on days 1, 4, and 7 was significantly lower in both septic and critically ill non-septic (CINS) patients compared to healthy subjects. Early increases in total TNF expression were associated with favorable discharge disposition and lower in-hospital mortality. ‘Immunocompetent’ endotype patients on day 1 had a higher proportion of favorable to non-favorable discharges compared to the ‘immunosuppressed’ endotype. Similarly, ‘immunocompetent’ endotype patients on day 4 had a higher in-hospital survival compared to the ‘immunosuppressed’ endotype patients. Finally, among septic patients, decreased total TNF and IFNγ expression were associated with 180-day mortality.

**Conclusions:**

Increased *ex vivo* whole blood TNF expression is associated with improved clinical outcomes. Further, the early ‘immunocompetent’ endotype is associated with favorable discharge and improved in-hospital and 180-day survival. The ability to functionally stratify septic patients based on blood cell function *ex vivo* may allow for identification of future immune modulating therapies.

## Introduction

Sepsis is defined as a dysregulated host immune response to infection leading to organ dysfunction (1, 2). The evolving definition of the septic state involves a spectrum of severity ranging from vital sign derangement to shock with end organ damage or failure (2–4). There is an increased prevalence of sepsis among high income countries, due in part to higher rates of comorbidities and immunocompromise (5, 6). Despite the increased incidence of sepsis, treatment remains largely supportive with the primary tenants of the Surviving Sepsis Best Practice Guidelines from 2021 focusing on early detection followed by resuscitation, hemodynamic and ventilatory support, and antimicrobial treatment (7). The immediate uncontrolled inflammation responsible for early shock or multiorgan failure has been attributed to both host innate and adaptive immune responses, dictated by the patient’s immunologic phenotype (8).

Complexity in treating sepsis stems from the heterogeneity of the host-specific phenotype and corresponding response. Such complexity leads to challenges in treating the physiologic sequalae of sepsis with immune modulation, requiring classification of septic patients according to their heterogenous immune responses (9). Current literature determines endotypes by the static timepoint of hospital admission, with less data available regarding immunologic status of patients throughout their clinical course (10–14). Endotype classification of a presenting septic patient has both prognostic value and broad therapeutic implications when determining candidates for immunomodulatory therapy.

Enzyme-Linked ImmunoSpot (ELISpot) Assay quantifies cellular production of cytokine in response to *ex vivo* stimulation. The use of different stimulants permits the quantification of both innate and adaptive immune responses, making it ideal for endotype classification (15, 16). Further, the ELISpot assay can be performed efficiently enough to be used in clinical decision-making (17). In this study, we tested the hypothesis that whole blood *ex vivo* functional TNF expression alone as well as an immunologic endotype based on both IFNγ and TNF expression could be used to identify clinical outcomes in sepsis patients. We further aimed to determine whether deriving an immunologic endotype based on both IFNγ and TNF expression was associated with clinical outcomes in septic patients.

## Methods

### Cohort selection

This is a secondary analysis of a prospective, observational study conducted at five U.S. tertiary care, academic medical centers (SPIES Consortium) (15). Patients were enrolled between February 2021 and July 2022. One hundred and seven patients admitted to the ICU with suspected sepsis (“septic”) and 68 patients admitted to the ICU without suspected sepsis (critically ill, non-septic; “CINS”) were included. Healthy control subjects were enrolled in the outpatient setting. Enrollment occurred across five clinical sites, each within a different healthcare system. Sepsis-3 criteria was used for sepsis definition (18). Diagnosis of sepsis versus non-sepsis was determined by site specific physician-investigators. Through this verification, 18 patients were recategorized from “septic” to “CINS”, and 6 were recategorized from “CINS” to “septic.” Specific exclusion criteria for healthy outpatient controls included current treatment with immunomodulators, anti-neoplastic therapies, or recent cancer diagnosis within six months. Given that the current work represents a secondary analysis of a previously published prospective, observational study, patient demographic information was included in the initial manuscript by Barrios et al. (15). Patient characteristics were similar across all three groups with the specific exceptions of the control cohort being disproportionately young and female, and the septic cohort having a significantly higher Charleston comorbidity index relative to the CINS cohort (15).

### Blood sampling and processing

Samples of heparinized whole blood labeled timepoint 1 (T1/day 1, collected 0-72 hours from admission), timepoint 2 (T2/day 4, collected 72-120 hours from admission), timepoint 3 (T3/day 7, collected 120-192 hours from admission) and timepoint 4 (T4/day 14, collected 312-360 hours or 13-15 days from admission) were collected. ELISpot assays were conducted with the TNF Immunospot kit (16). Briefly, *ex vivo* activation of 5µL of whole blood was achieved via lipopolysaccharide (LPS from *E. coli*, serotype O55:B5 at 1.25 ng/mL, ENZO Life Sciences, Famingdale, NY) treatment for a duration of 22 hours ± 10 minutes. ELISpot assay was initiated within one hour of blood draw, with blood kept at room temperature on a rocker table until processing was initiated. Samples were analyzed using a CTL S6 Entry or S6 FluoroCore™ ELISpot reader utilizing identical instrument settings and protocols at each clinical site. Whole blood IFNγ expression in response to *ex vivo* anti-CD3/CD28 mAb stimulation was also conducted simultaneously on each sample with results reported previously (15). All samples were analyzed in duplicate. Quantitative outputs included the number of spot-forming units (SFU) which represents the number of cells per 5 µl whole blood sample that secrete the cytokine in question, spot size (*μ*m^2^; SS) which represents the amount of cytokine produced per stimulated cell, and total expression (*μ*m^2^; TE) which serves as a combined metric of spot-forming units and spot size, using the Immunospot^®^ SC software suite version 7.0.30.4 (ImmunoSpot, Cleveland, OH). SFUs are representative of individual blood cells producing TNF. SS serves as a quantitative measure of TNF produced per cell.

Additional laboratory analyses included whole blood total leukocyte and absolute monocyte/PMN counts on EDTA-anticoagulated whole blood utilizing facility-specific Clinical and Diagnostics Laboratory or a research Beckman-Coulter Dx500 or Dx900 hemocytometer 24 (Beckman-Coulter, Brea, CA). Cytokine and additional plasma protein analyses, including serum soluble programmed death-ligand 1 (sPD-L1) were conducted at the University of Florida Sepsis and Critical Illness Research Center (SCIRC) utilizing the Luminex MagPix^®^ platform (Bio-Rad, Hercules, CA).

### Clinical outcome data collection

The primary outcome of the study was 180-day mortality, which was verified by clinical records, telephone follow-up with either the patient or designated contact, and the US Social Security Death Index. Secondary outcomes included all-cause in-hospital (30-day mortality and unfavorable discharge disposition (defined as discharge to skilled nursing facility, long-term acute care facility, or hospice). Clinical data collection was performed at each clinical site and entered into Research Electronic Data Capture (REDCap) software (REDCap, Nashville, TN) and laboratory testing endpoints entered into an electronic case report form (eCRF) by approved research staff (REDCap™, Vanderbilt University). Data were managed primarily by research team members at the University of Florida Clinical and Translational Science Institute (CTSI). Results from blood sampling and processing were also entered into the eCRF including ELISpot, total leukocyte counts, absolute monocyte/PMN counts, and plasma protein and cytokine concentration data.

### Endotyping patients by ELIspot

Four patient endotypes were defined prospectively in the sepsis and CINS cohorts based on their initial (T1) ELIspot results. Specifically, patients were assigned either an elevated (+) or suppressed (-) ELIspot if the total expression (TE) was greater or less than the median value obtained from healthy subjects, respectively. Patients who had both increased LPS-stimulated TNF and anti-CD3/CD28 mAb-stimulated IFN-γ expression compared to healthy subjects (TNF^+^/IFNγ^+^) were defined as being ‘immune competent’. In contrast, patients with *ex vivo* TNF and IFNγ expression below median values from healthy subjects (TNF^-^/IFNγ^-^) were defined as having an ‘immunosuppressed’ endotype. Patients with one of two ‘mixed’ endotypes had *ex vivo* TNF or IFNγ expression that was either greater and less than (TNF^+^/IFNγ^-^), or less than and greater than (TNF^-^/IFNγ^+^) median values from healthy subjects.

### Statistical analysis

Statistical analysis included Fisher’s exact (categorical variables) and Mann-Whitney or Kruskal-Wallis ANOVA tests (continuous variables) as indicated. Area under the receiver operating characteristics curve (AUROC) values with 95% confidence intervals (computed with 2000 stratified bootstrap replicates) were used to assess discrimination. Multivariate Cox regressions were performed to construct a combination of metrics, and then the combined metric was assessed for improved overall performance. *Post hoc* tests were performed for continuous outcomes using the Dunn test. For *post hoc* analyses of categorical outcomes, separate 2 × 2 Fisher’s exact tests were performed. All significance tests were 2-sided, with a raw p value of less than or equal to 0.05 were considered statistically significant. Analyses were performed using the R Project statistical package, version 4.1.0 (R Project for Statistical Computing, Vienna, Austria). Data is reported as mean ± SD.

## Results

### LPS-stimulated TNF expression measured as SFU and SS

**Figure 1:**
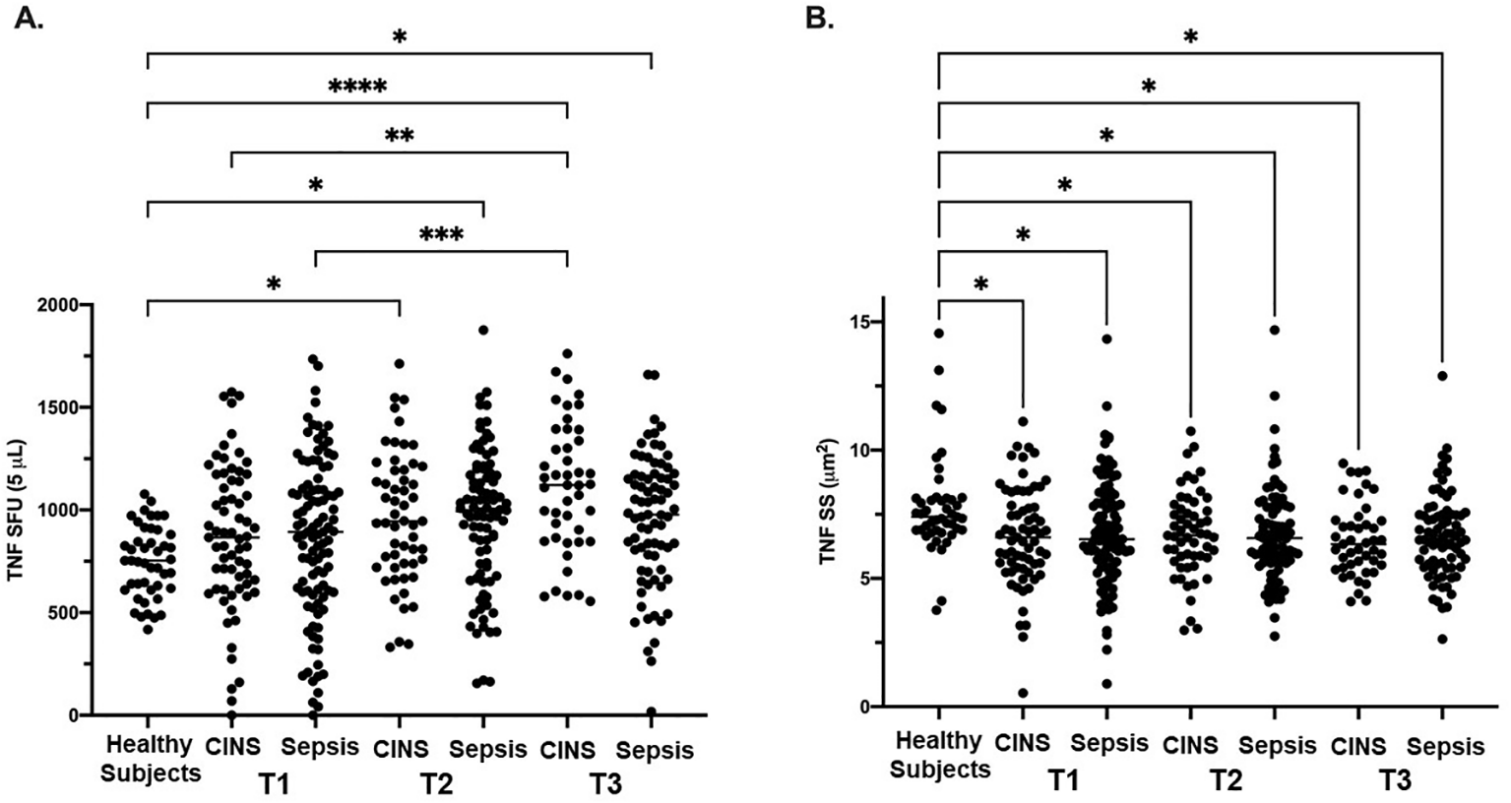
LPS-stimulated TNF expression as determined by ELISpot spot forming units (SFU) and spot size (SS) in Sepsis, and CINS patients at timepoints 1, 2, and 3 following ICU admission, and in healthy control subjects. Values represent mean and individual subject responses for TNF SFU **(A)** and SS **(B)** at T1, T2, and T3. T1: healthy cohort n=45, CINS n=67, septic n=103; T2: healthy cohort n=45, CINS n=55, septic n=90; T3: healthy cohort n=45, CINS n=45, septic n=77. *P < 0.05, **P < 0.01, ***P < 0.001, ****P < 0.0001, as determined by Kruskal-Wallis ANOVA and *post hoc* analyses using Dunn’s test. SFU, spot-forming units.

There were no significant differences noted in TNF SFU when comparing the septic and CINS cohorts to the healthy subject cohort at timepoint 1 (0-72 hrs). (**Figure 1A**) At timepoint 2 (73-120 hrs), compared with the healthy cohort, both the septic (956 ± 339 vs. 747 ± 172, p<0.001) and CINS (965 ± 318 vs. 747 ± 172, p<0.01) patient cohorts demonstrated a significantly higher number of TNF SFU compared to healthy subjects. (**Figure 1B**) At timepoint 3 (121-192 hrs), compared with the healthy cohort, both the septic (950 ± 320 vs. 747 ± 172, p<0.001) and CINS (1112 ± 317 vs. 747 ± 172, p<0.0001) patient cohorts demonstrated a significantly higher number of TNF SFU compared to healthy donors. Given that SFU represents a quantitative measurement of TNF-producing cells, this data supports an increase in cells secreting TNF in CINS and septic patients at the T1 and T2 timepoints.

At timepoint 1, both the septic (6.7 ± 2.1 *μ*m^2^ vs. 7.8 ± 1.9 *μ*m^2^, p<0.01) and CINS (6.6 ± 1.9 *μ*m^2^ vs. 7.8 ± 1.9 *μ*m^2^, p<0.01) patient cohorts demonstrated a significantly smaller TNF SS compared to the control cohort. At timepoint 2, both the septic (6.7 ± 1.8 *μ*m^2^ vs. 7.8 ± 1.9 *μ*m^2^, p<0.001) and CINS (6.7 ± 1.7 *μ*m^2^ vs. 7.8 ± 1.9 *μ*m^2^, p<0.01) patient cohorts demonstrated a significantly smaller TNF SS compared to the control cohort. At timepoint 3, both the septic (6.6 ± 1.7 *μ*m^2^ vs. 7.8 ± 1.9 *μ*m^2^, p<0.001) and CINS (6.5 ± 1.4 *μ*m^2^ vs. 7.8 ± 1.9 *μ*m^2^, p<0.001) patient cohorts demonstrated a significantly smaller TNF SS compared to the control cohort. Given that SS represents a quantitative measurement of TNF produced per cell, this data supports decreased TNF production per cell in CNS and septic patients at the T1, T2, and T3 timepoints.

### Correlation of LPS-stimulated total TNF expression with discharge and mortality

**Figure 2:**
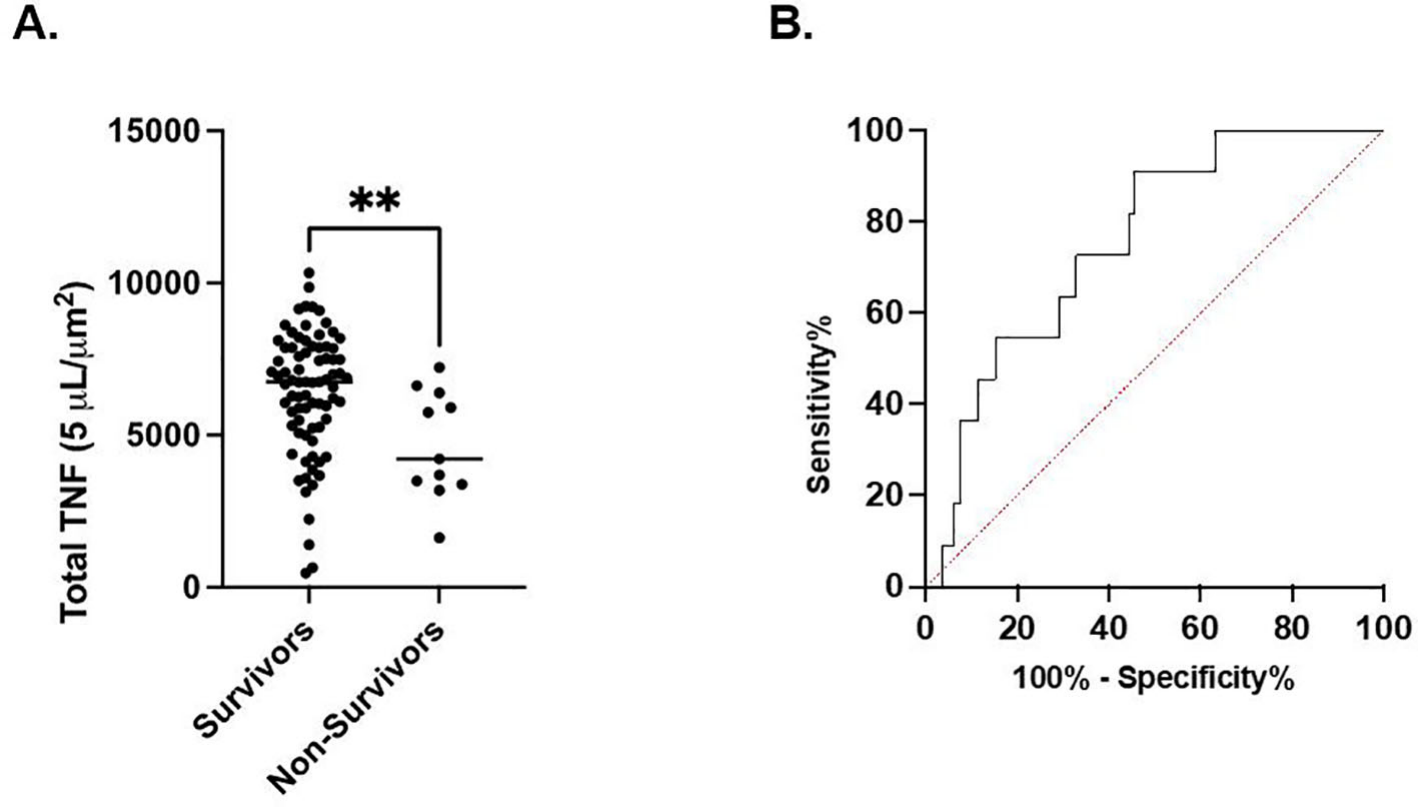
LPS-stimulated total TNF expression at timepoint 2 is associated with in-hospital mortality. **(A)**. Comparison of in-hospital mortality of the septic patient cohort. **(B)** Area under the Receiver Operator Curve (AUROC) for total LPS-stimulated TNF expression in differentiating in-hospital mortality. **P < 0.01, as determined by unpaired Mann-Whitney test. AUROC=0.7572, p=0.0059.

In septic patients examined at timepoint 1, total TNF expression was not different between septic patients who survived 180 days versus those who expired. However, at timepont 2, total TNF expression was significantly higher in the cohort of patients characterized by 180-day survival compared to the cohort characterized by in-hospital mortality (6394 ± 2026 *μ*m^2^ vs. 4682 ± 1779 *μ*m^2^, p<0.01). (**Figure 2A**) Receiver Operating Characteristic curve demonstrated a significant association between survival to discharge and total TNF expression measured at timepoint 2 (AUC=0.76, p<0.01). (**Figure 2B**) At timepoint 3, total TNF expression was significantly higher in the cohort of patients characterized by survival to discharge compared to the cohort characterized by in-hospital mortality (6480 ± 2122 *μ*m^2^ vs. 4756 ± 2384 *μ*m^2^, p=0.03). At timepoint 3, total TNF expression was significantly higher in the cohort of patients who survived to discharge compared to those who suffered in-hospital mortality (648 ± 2122 *μ*m^2^ vs. 4756 ± 2384 *μ*m^2^, p=0.03). Receiver Operating Characteristic curve demonstrates a significant association between survival to discharge and total TNF expression measured at timepoint 2 (AUC=0.72, p=0.04). No significant difference was noted in total TNF expression at timepoints 1 or 4 between the survival to discharge and in-hospital mortality cohorts. (**Supplementary Table 1**) No significant associations in total TNF expression at timepoints 1 or 4 and in-hospital mortality were noted by ROC curve. (**Supplementary Table 2**).

**Figure 3:**
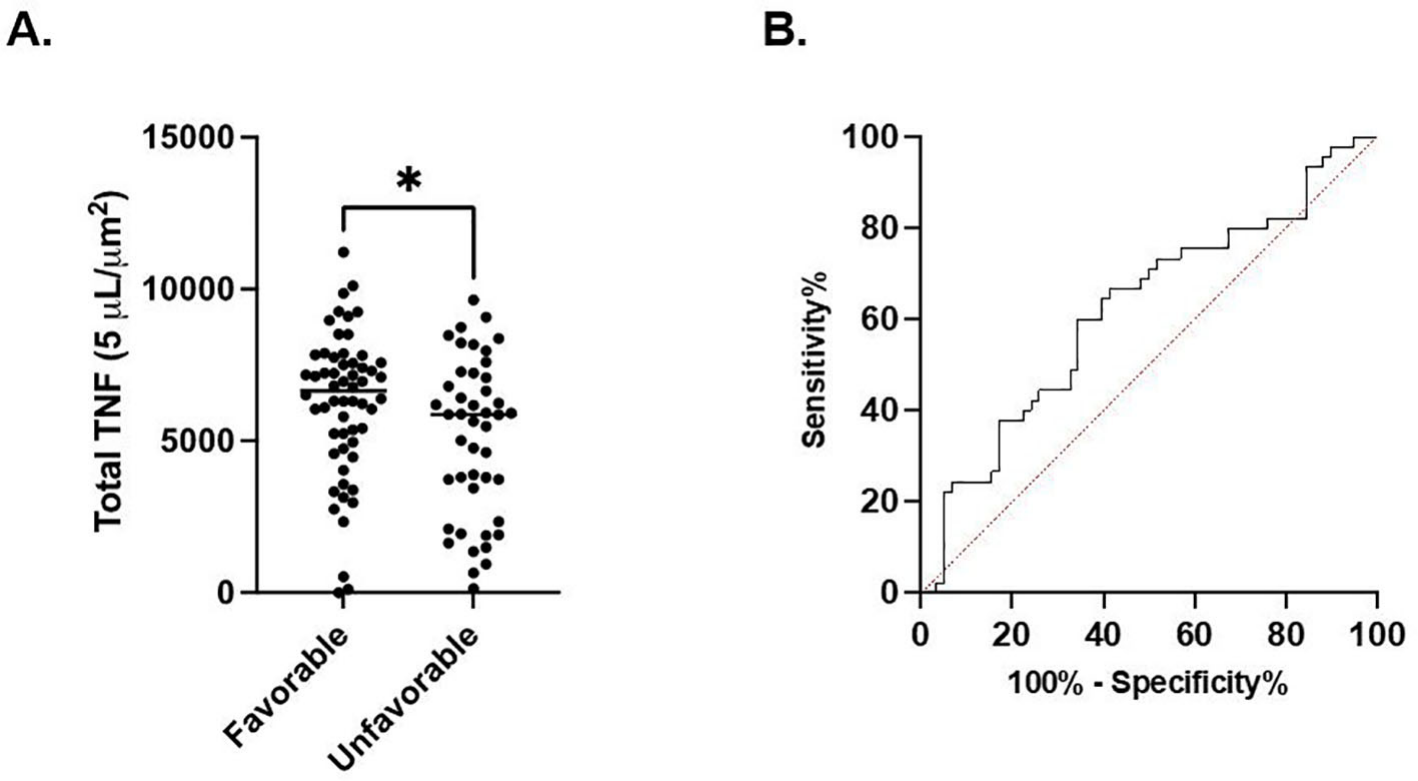
Total LPS-stimulated TNF expression at timepoint 1 is associated with discharge. **(A)**. Comparison of favorable and non-favorable discharge patient populations in the septic patient cohort. **(B)** Area under the Receiver Operator Curve (AUROC) for total LPS-stimulated TNF expression in differentiating favorable vs. unfavorable discharge. *P < 0.05, as determined by unpaired Mann-Whitney test. AUROC curve=0.6218, p=0.0345.

In septic patients examined at timepoint 1, total TNF expression was significantly higher in the cohort of patients characterized by a favorable discharge compared to the cohort with an unfavorable discharge (6209 ± 2380 *μ*m^2^ vs. 5115 ± 2578 *μ*m^2^, p=0.03). (**Figure 3A**) Receiver Operating Characteristic curve demonstrated a significant association between discharge favorability and total TNF expression measured at timepoint 1 (AUC=0.62, p=0.03). (**Figure 3B**). No significant difference in total TNF expression at timepoints 2, 3, or 4 between the favorable and unfavorable discharge cohorts was noted.

### Endotype classification associations with demographic, clinical, and serum differences

Receiver Operating Characteristic curves utilizing a Cox regression at timepoint 2 were stratified into endotypes of septic patients based on their TNF and IFNg total expression (TE). In septic patients whose total TNF and IFNγ expression was below the median value from healthy subjects, they were designated as being ‘immunosuppressed’ (TNFα^-^/IFNγ^-^), for septic patients whose TE was above the median TNF and IFNγ TE from healthy subjects were defined as being ‘immune competent’. Finally, for septic patients who had either TNF or IFNγ total expression above the median for healthy subjects, with the other below the median for healthy subjects (TNF^-^/IFNγ^+^, TNF^+^/IFNγ^-^), they were defined as a ‘mixed’ endotype. Fisher’s exact test demonstrated a significant difference in differential discharge rates across the four endotypes at timepoint 1 (p=0.01). (**Supplementary Figure 1A**) Patients with th*e* ‘immune competent’ endotype (TNF^+^/IFNγ^+^) were significantly younger compared to patients with the immunosuppressed endotype (TNF^-^/IFNγ^-^) (52 ± 15 years vs. 63 ± 15 years, p=0.0461). (**Supplementary Figure 1B**) Patients with the ‘immune competent’ (TNF^+^/IFNγ^+^) endotype had a significantly lower T1 SOFA score compared to ‘immunosuppressed’ (TNF^-^/IFN-γ^-^) endotype at timepoint 1 (4.4 ± 3.1 vs. 7.7 ± 4.2, p=0.0063). (**Supplementary Figure 1C**) Similarly patients with the ‘immune competent’ endotype had significantly lower serum sPD-L1 compared to ‘immunosuppressed’ endotype at timepoint 1 (209.3 ± 173.9 pg/mL vs. 424.2 ± 452.0 pg/mL, p=0.0366). (**Supplementary Figure 1D**).

Fisher’s exact test demonstrated a significant difference with regard to in-hospital mortality across the four endotypes at timepoint 2 (p<0.01). (**Supplementary Figure 2A**). Patients with the ‘immune competent’ endotype had a significantly lower T2 SOFA score compared to the ‘immunosuppressed’ endotype at timepoint 2 (3.2 ± 3.0 vs. 6.3 ± 4.0, p=0.0113). (**Supplementary Figure 2B**) Patients with the ‘immune competent’ endotype also had significantly lower plasma IFNγ concentrations compared to patients with the ‘immune suppressed’ endotype at timepoint 2 (48 ± 20 pg/mL vs. 65 ± 36 pg/mL, p=0.0499). (**Supplementary Figure 2C**) Finally, patients with the ‘immune competent’ endotype had significantly lower plasma TNF concentrations compared to patients with the ‘immunosuppressed’ endotype at timepoint 2 (20 ± 13 pg/mL vs. 29 ± 13 pg/mL, p=0.0372). (**Supplementary Figure 2D**).

**Figure 4:**
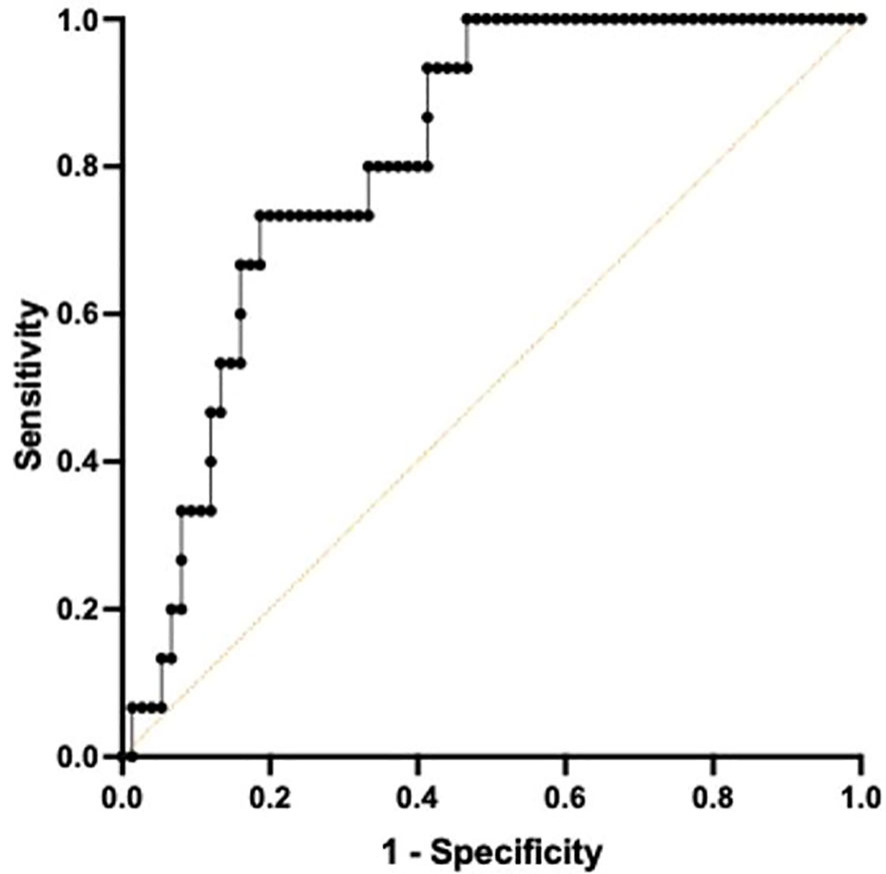
Area under the Receiver Operator Curve (AUROC) using both TNF^+^ and IFNγ^+^ on day 4 following ICU admission in differentiating 180-day mortality. A multiple Cox regression to predict survival time using both total TNF and IFNγ was conducted to construct a combined metric. When predicting 180-day mortality using the constructed metric, AUROC curve was 0.8133, 95% confidence interval was 0.7173-0.9094. p=0.0001.

Receiver Operating Characteristic curve utilizing a combined metric constructed by a Cox regression with TNF and IFNγ at T2 demonstrated high predictive quality in determining 180-day mortality with an AUC of 0.813 (CI 0.7173-0.9094, p<0.001). (**Figure 4**) The sensitivity for predicting 180-day mortality is 0.800 while the specificity is 0.667.

## Discussion

### Key findings

This prospective, multi-center observational study has demonstrated that the host immune response to critical illness, as defined by *ex vivo* whole blood production of TNF, varied in response to critical illness (**Figures 1**, **2**) and was associated with long-term clinical outcomes including discharge disposition and in-hospital mortality (**Supplementary Figures 1**, **2**). Whole blood stimulated *ex vivo* with LPS demonstrated increased TNF production in both septic and CINS cohorts when compared to healthy subjects.

There was time-dependent variation in TNF production in both septic and CINS cohorts, with increased numbers of TNF-producing cells in both the septic and CINS cohorts at day 4 and 7 compared to the healthy subjects (**Figure 1**). Interestingly, the amount of TNF produced per leukocyte was greater in the healthy subjects compared to the septic and CINS cohorts at all timepoints out to seven days (**Figure 1**). When considering clinical outcomes, TNF expression measured at day 1 was higher in patients who ultimately had a favorable discharge disposition compared to those patients with an unfavorable discharge disposition. Further, total TNF expression measured at day 4 was higher in patients who survived to discharge when compared to the in-hospital mortality cohort. (**Supplementary Figures 1**, **2**).

Endotypes were established as described herein. Differential discharge rates were statistically different between endotype cohorts at day 1 and mortality was different between endotype cohorts at day 4. The ‘immune competent’ endotype cohort (TNF^+^/IFNγ^+^) was significantly younger than both the ‘immunosuppressed’ (TNF^-^/IFNγ^-^) and one of the ‘mixed’ (TNF^+^/IFNγ^-^) cohorts and had a lower SOFA score than the ‘immunosuppressed’ endotype at day 1. The ‘immune competent’ cohort had a lower SOFA score than the immunosuppressed cohort at day 4. The immune competent cohort also had lower plasma sPD-L1 at day 1, as well as lower plasma IFNγ and TNF at day 4 compared to the immunosuppressed cohort at the same respective timepoint. Finally, total TNF and IFN-γ expression at day 4 demonstrated high predictive quality in determining 180-day mortality (**Figure 4**).

### Context

ELISpot is a well-established tool with the capability to assess immunologic function in various pathologic conditions (19–22) and has been specifically used to assess the immunologic status of the septic and critically-ill patients (16, 23–25). Our multi-institutional consortium has previously utilized ELISpot to characterize patients based on *ex vivo* IFNγ production following targeted T cell receptor stimulation and the relationship of the subsequent immunologic phenotype to clinical outcomes (26). The present study utilizes ELISpot analysis with *in vitro* measurement of LPS-stimulated TNF as an additional metric of host immune activation within the same cohort of prospectively collected human whole blood samples. While previous studies have utilized ELISpot to quantify cell activation and secretion of TNF in response to LPS treatment, they have done so in either highly enriched polymorphonuclear cells (PMN), peripheral blood mononuclear cells (PBMC), or isolated B cells (27–29). Previously, our group compared the ability of the whole blood ELISpot with a more traditional ELISpot assay using PBMCs in sepsis. IFNγ and TNF ELISpot assays on whole blood and PBMCs were undertaken in control, critically ill non-septic, and septic patients. Whole blood ELISpot was easy to perform, and results were generally comparable to PBMC-based ELISpot. However, the whole blood ELISpot assay revealed that non-monocyte, myeloid populations are a significant source of ex vivo TNF production (16). As in our previous study, then, a major advantage of the current approach has been the use of whole blood (rather than a single isolated cell type), which allows for the assessment of an integrated immunologic function from all blood components including leukocytes, erythrocytes, platelets, plasma proteins, and metabolites.

Clinical sepsis is known to involve the release and propagation of circulating proinflammatory cytokines, including TNF, early in the septic process (30). The mechanism by which TNF contributes to the global milieu of sepsis and how it may relate to clinical outcomes, however, remains ambiguous. Several studies have implicated single nucleotide polymorphisms of the TNF coding sequence as a potential protective factor against severe sepsis (30, 31). While the use of laboratory testing to quantify TNF has demonstrated efficacy as a screening tool for clinical sepsis, it is not routinely used in a diagnostic or prognostic capacity (32). In the present study, we utilized a diluted whole blood ELISpot assay to quantify both the number of TNF-producing cells (represented by SFU) and amount of TNF released per cell (represented by SS). In doing so, we demonstrated that the amount of TNF released per cell was lower in the septic and CINS patients than in the control cohort at the 1-, 4-, and 7-day post-admission timepoints. This finding is concordant with and may potentially be explained by previous literature describing the phenomenon of “immunoparalysis” in sepsis, where lymphocyte apoptosis and endotoxin tolerance in surviving lymphocytes results in decreased overall secretion of inflammatory cytokines including TNF and IFNγ (33–35). Interestingly, the number of TNF-releasing cells as measured by SFU was elevated in septic and CINS patients at both the 4- and 7-day post-admission timepoints. Though this may seem to contradict the principle of immunoparalysis, the decreased SS and increased SFU in septic and CINS patients may represent a higher degree of endotoxin tolerance and lower contribution from lymphocyte apoptosis as measured by ELISpot in this analysis. Finally, in considering total patient TNF, we found higher TNF at the day-1 post-admission timepoint is associated with a favorable discharge disposition and higher TNF at the day-4 post-admission timepoint is associated with improved survival to discharge. These data further support the proposition that TNF quantified by ELISpot holds the potential for prognostic use in septic and critically-ill patients and that patients less phenotypically inclined to immunoparalysis may have superior clinical outcomes.

The complexity of clinical sepsis and diversity of immunologic endotypes between patients leads to challenges in treatment (9). As previously outlined, these different endotypes may result in variation in cytokine release by way of differences in either lymphocyte apoptosis or endotoxin tolerance, culminating in significant variation in clinical outcomes (33–35). Given the heterogenous nature of the immunologic response to sepsis, it may be beneficial to classify patients in order to target treatment. Previous classification systems grouped patients by plasma concentration of immunologic markers, including TNF, interleukin-6 (IL-6), and IL-8 at the time of hospital admission (10–12). Subsequent literature has utilized transcriptomic data from patient leukocytes and sorted into endotypes named Sepsis Response Signatures (SRS) 1 and 2, noting differences in relative immunosuppression and mortality profile between the groups (13). Finally, the Molecular Diagnosis and Risk Stratification of Sepsis (MARS) consortium utilized genome-wide blood gene expression profiles to group patients into endotypes MARS1-4 for adult patients and MARS1, 2, and 4 for pediatric patients. This endotyping system included more granularity in regard to immunosuppression of the innate, adaptive or both host immune responses (14). Current endotype systems have utilized the single timepoint of hospital admission rather than incorporating repeat testing, which more accurately reflects the dynamic physiology of the critically-ill patient. The present study utilized ELISpot data from patient whole blood, including total TNF and IFNγ expression to group patients into TNF high low, and IFNγ high (+) or low (-). The cutoff values were established as the median value from a population of healthy controls (**Supplementary Figure 3**). We developed a novel endotype method based on peripheral whole blood sampling, which is easily repeatable at timepoints throughout a patient’s clinical course. This endotype nomenclature combines the predictive ability of two unique components of a patient’s immunologic phenotype, with each patient classified as being ‘immunosuppressed’ (TNF^-^/IFNγ^-^), ‘mixed’ (TNF^+^/IFNγ^-^, TNF^-^/IFNγ^+^), or ‘immune competent’ (TNF^+^/IFNγ^+^). The two distinct “mixed” endotype groups were maintained as separate groups given the potential prognostic value held by each in representing a competent function of each specific immunologic endotype.

Following stratification by endotype, we identified a significant difference in both favorable discharge disposition and in-hospital mortality by endotype group at ICU admission days 1 and 4, respectively. Further, we noted that patients in the ‘immune competent’ cohort on ICU admission day 1 were younger and had a lower SOFA score when compared to those in the ‘immunosuppressed’ cohort. A lower SOFA score was also noted in the ‘immune competent’ cohort compared to the ‘immunosuppressed’ cohort when endotype was completed on ICU admission day 4. These data reinforce that patients with less immunosuppression may belong to a younger population and have less organ-failure related morbidity throughout their clinical course. Additionally, plasma sPD-L1 was found to be significantly higher in the ‘immunosuppressed’ cohort compared to the ‘immune competent’ cohort on ICU admission day 1. sPD-L1 has been demonstrated in cancer cells to serve as a marker for cellular exhaustion following prolonged cytokine exposure, similar in nature to the principle of immunoparalysis (36, 37). Therefore, higher sPD-L1 levels in the ‘immunosuppressed’ cohort supports a possible endotypic propensity toward immunosuppressed in these patients. Interestingly, plasma concentrations of both TNF and IFNγ were higher in the ‘immunosuppressed’ cohort compared to the ‘immune competent’ cohort on ICU admission day 4. While these data may seem immediately contrary to what we would expect based on endotype classification, this finding is concordant with the current understanding of septic immunosuppression and our previously reported increased levels of sPD-L1 in the ‘immunosuppressed’ cohort. As discussed previously, a key component of immunosuppression seen in the septic patient is leukocyte exhaustion (34). Given that our ‘immunosuppressed’ cohort was defined by a lower *ex vivo* response to leukocyte stimulation following whole blood collection, a potential mechanism for such a perceived discrepancy is a higher propensity toward leukocyte exhaustion *in vivo* prior to sample collection in the ‘immunosuppressed’ cohort. Higher levels of systemic circulating cytokines in patients with an ‘immunosuppressed’ endotype, then, could possibly be a contributor to leukocyte exhaustion and immunosuppression, as measured by *ex vivo* ELISpot assay, rather than a downstream effect of immunologic activity. This proposed mechanism is supported by increased leukocyte exhaustion as measured by sPD-L1 in the ‘immunosuppressed’ cohort. Such a mechanism is speculative based on the current data and requires further study with titration of specific immunologic stimulation in patients of each endotype classification.

There are limitations to our study that require discussion. Sample size was limited despite multicenter enrollment given improved outcomes of septic and critically-ill patients over the past two decades (38, 39). As with our previous study, discriminatory analysis was completed only on the septic patient cohort given the exceedingly low 180-day and in-hospital mortality rates of the CINS patients (4% and 1%, respectively). Another limitation of our study was the disproportionately younger age and female predominance of the healthy (control) cohort compared to the septic and CINS patent cohorts, despite matching efforts. However, the median age of the control study was above 45 years, an age above which incidence of more adverse outcomes have been shown to occur in critically-ill patients (38). Finally, while the present study represents a novel method of patient classification based on endotypes with the potential for prognostication, the precise cellular signalling mechanisms underlaying immune competency and immunosuppression as described by the ELISpot assay require further research.

## Data Availability

The raw data supporting the conclusions of this article will be made available by the authors, without undue reservation.

## Supplementary material

Supplementary Table 1 Total TNF expression at timepoints 1-4 and association with favorable discharge. AUROC, Area Under Receiver Operator Characteristic, ROC, Receiver Operator Characteristic, F, favorable, NF, Non-favorable.

Supplementary Table 2 Total TNF at timepoints 1-4 and association with in-hospital mortality. AUROC, Area Under Receiver Operator Characteristic, ROC, Receiver Operator Characteristic, S, Survivor (in-hospital), NS, Non-survivor.

Supplementary Figure 1 ‘Immune competent’ (TNF^+^/IFNγ^+^), ‘immunosuppressed’ (TNF^-^/IFNγ^-^) and ‘mixed’ (TNF^-^/IFNγ^+^, TNF^+^/IFNγ^-^) endotypes, determined at timepoint 1 are stratified by predictive, physiologic, and biomarker indices. Above (+) or below (-) represent individual values obtained from the patients when compared to the median values from the healthy subject. **(A)** Values represent favorable vs. non-favorable discharge. A Fisher’s exact test to differentiate discharge using the identified four endotypes was conducted and demonstrated that the differential discharge rates across four is statistically significant (p=0.0109). Patient values of **(B)** age (years), **(C)** SOFA score on timepoint 1 following ICU admission, and **(D)** serum sPD-L1 on timepoint 1 following ICU admission (reported as pg/mL). For **(B-D)**, *P < 0.05, **P < 0.01, as determined by Kruskal-Wallis ANOVA and *post hoc* analyses using Dunn’s test. SOFA, sequential organ failure assessment.

Supplementary Figure 2 ‘Immune competent’ (TNF^+^/IFNγ^+^), ‘immunosuppressed’ (TNF^-^/IFNγ^-^) and ‘mixed’ (TNF^-^/IFNγ^+^, TNF^+^/IFNγ^-^) endotypes, on day 4 following ICU admission are stratified by predictive, physiologic, and biomarker indices. Above (+) or below (-) represent individual values obtained from the patients when compared to the median values from the healthy subject. **(A)** Values represent in-hospital mortality. A Fisher’s exact test to differentiate discharge using the identified four endotypes was conducted and demonstrated that the differential discharge rates across four is statistically significant (p=0.0120). **(B)** Timepoint 2 SOFA score, **(C)** Serum IFNγ at timepoint 2 (reported as pg/mL), and **(D)** Serum TNFα on timepoint 2 following ICU admission (reported as pg/mL). **P < 0.01 as determined by Kruskal-Wallis ANOVA and *post hoc* analyses using Dunn’s test. SOFA, sequential organ failure assessment.

Supplementary Figure 3 Median total TNF **(A)** and IFNγ **(B)** levels within a healthy control population; utilized in order to establish A) TNF^+/-^ and B) IFNγ^+/-^ endotypes. Values represent mean median **(A)** TNF SFU and B) IFNγ SFU for health controls. Healthy cohort n=45. SFU, spot-forming units.
