# Supplementary material for: Surviving septic patients endotyped with a functional assay demonstrate active immune responses
Source: Front Immunol. 2024 Oct 14;15:1418613. doi: 10.3389/fimmu.2024.1418613 (PMC11513262; doi:10.3389/fimmu.2024.1418613)
Supplement: Supplementary Table 1 — Total TNF expression at timepoints 1-4 and association with favorable discharge. AUROC, Area Under Receiver Operator Characteristic, ROC, Receiver Operator Characteristic, F, favorable, NF, Non-favorable. [file Table1.docx]

Supplemental Table 1: Total TNF association with in-hospital mortality by time point

Discharge:

|  | AUROC | ROC Curve p Value | # Favorable | # Non-Favorable | Trend |
| --- | --- | --- | --- | --- | --- |
|  |  |  |  |  |  |
| T1 Total TNF | 0.6218 | **0.0345** | 58 | 45 | F>NF |
| T2 Total TNF | 0.5938 | 0.1289 | 51 | 39 | F>NF |
| T3 Total TNF | 0.5540 | 0.4177 | 43 | 34 | F>NF |
| T4 Total TNF | 0.5818 | 0.3646 | 22 | 20 | F>NF |

Supplemental Table 2: Total TNF association with discharge by time point

Mortality:

|  | AUROC | ROC Curve p Value | # Survivors | # Non-Survivor | Trend |
| --- | --- | --- | --- | --- | --- |
|  |  |  |  |  |  |
| T1 Total TNF | 0.6284 | 0.1236 | 89 | 14 | S>NS |
| T2 Total TNF | 0.7572 | **0.0059** | 79 | 11 | S>NS |
| T3 Total TNF | 0.7157 | **0.0364** | 68 | 9 | S>NS |
| T4 Total TNF | 0.5526 | 0.7318 | 38 | 4 | S=NS |
