# Supplementary figures and images for: Surviving septic patients endotyped with a functional assay demonstrate active immune responses
Source: Front Immunol. 2024 Oct 14;15:1418613. doi: 10.3389/fimmu.2024.1418613 (PMC11513262; doi:10.3389/fimmu.2024.1418613)

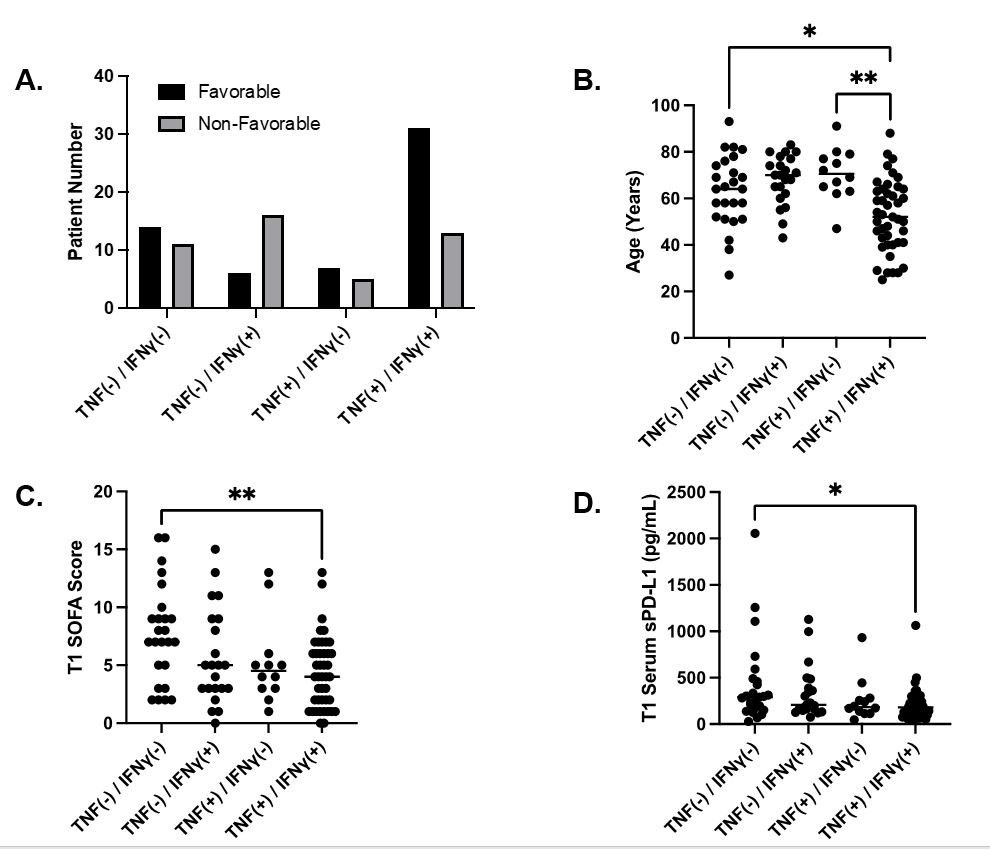

Supplement: Supplementary Figure 1 — ‘Immune competent’ (TNF+/IFNγ+), ‘immunosuppressed’ (TNF-/IFNγ-) and ‘mixed’ (TNF-/IFNγ+, TNF+/IFNγ-) endotypes, determined at timepoint 1 are stratified by predictive, physiologic, and biomarker indices. Above (+) or below (-) represent individual values obtained from the patients when compared to the median values from the healthy subject. (A) Values represent favorable vs. non-favorable discharge. A Fisher’s exact test to differentiate discharge using the identified four endotypes was conducted and demonstrated that the differential discharge rates across four is statistically significant (p=0.0109). Patient values of (B) age (years), (C) SOFA score on timepoint 1 following ICU admission, and (D) serum sPD-L1 on timepoint 1 following ICU admission (reported as pg/mL). For (B-D), *P < 0.05, **P < 0.01, as determined by Kruskal-Wallis ANOVA and post hoc analyses using Dunn’s test. SOFA, sequential organ failure assessment. [file Image1.jpeg]

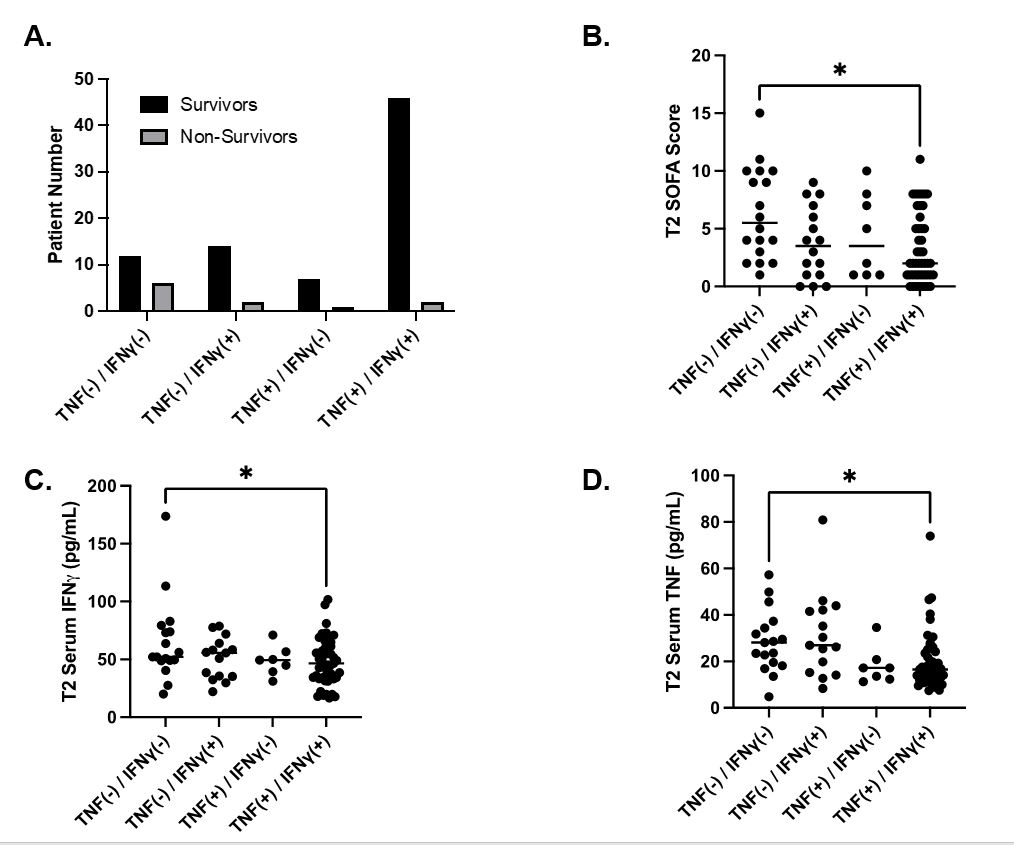

Supplement: Supplementary Figure 2 — ‘Immune competent’ (TNF+/IFNγ+), ‘immunosuppressed’ (TNF-/IFNγ-) and ‘mixed’ (TNF-/IFNγ+, TNF+/IFNγ-) endotypes, on day 4 following ICU admission are stratified by predictive, physiologic, and biomarker indices. Above (+) or below (-) represent individual values obtained from the patients when compared to the median values from the healthy subject. (A) Values represent in-hospital mortality. A Fisher’s exact test to differentiate discharge using the identified four endotypes was conducted and demonstrated that the differential discharge rates across four is statistically significant (p=0.0120). (B) Timepoint 2 SOFA score, (C) Serum IFNγ at timepoint 2 (reported as pg/mL), and (D) Serum TNFα on timepoint 2 following ICU admission (reported as pg/mL). **P < 0.01 as determined by Kruskal-Wallis ANOVA and post hoc analyses using Dunn’s test. SOFA, sequential organ failure assessment. [file Image2.jpeg]

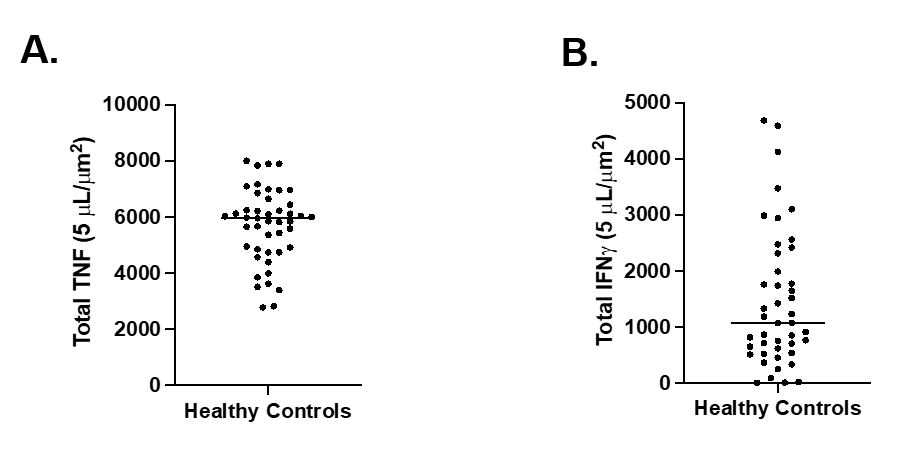

Supplement: Supplementary Figure 3 — Median total TNF (A) and IFNγ (B) levels within a healthy control population; utilized in order to establish A) TNF+/- and B) IFNγ+/- endotypes. Values represent mean median (A) TNF SFU and B) IFNγ SFU for health controls. Healthy cohort n=45. SFU, spot-forming units. [file Image3.jpeg]
